# Calcium‐regulated mitochondrial ATP‐Mg/P_i_ carriers evolved from a fusion of an EF‐hand regulatory domain with a mitochondrial ADP/ATP carrier‐like domain

**DOI:** 10.1002/iub.1931

**Published:** 2018-10-03

**Authors:** Steven P. D. Harborne, Edmund R. S. Kunji

**Affiliations:** ^1^ School of Biomedical Sciences and Astbury Centre for Structural Molecular Biology University of Leeds Leeds LS2 9JT UK; ^2^ Medical Research Council Mitochondrial Biology Unit University of Cambridge Cambridge CB2 0XY UK

**Keywords:** transport, mitochondrion, metabolic energy generation, translocase, translocator, mitochondrial carrier, evolution

## Abstract

The mitochondrial ATP‐Mg/P_i_ carrier is responsible for the calcium‐dependent regulation of adenosine nucleotide concentrations in the mitochondrial matrix, which allows mitochondria to respond to changing energy requirements of the cell. The carrier is expressed in mitochondria of fungi, plants and animals and belongs to the family of mitochondrial carriers. The carrier is unusual as it consists of three separate domains: (i) an N‐terminal regulatory domain with four calcium‐binding EF‐hands similar to calmodulin, (ii) a loop domain containing an amphipathic α‐helix and (iii) a mitochondrial carrier domain related to the mitochondrial ADP/ATP carrier. This striking example of three domains coming together from different origins to provide new functions represents an interesting quirk of evolution. In this review, we outline how the carrier was identified and how its physiological role was established with a focus on human isoforms. We exploit the sequence and structural information of the domains to explore the similarities and differences to their closest counterparts; mitochondrial ADP/ATP carriers and proteins with four EF‐hands. We discuss how their combined function has led to a mechanism for calcium‐regulated transport of adenosine nucleotides. Finally, we compare the ATP‐Mg/P_i_ carrier with the mitochondrial aspartate/glutamate carrier, the only other mitochondrial carrier regulated by calcium, and we will argue that they have arisen by convergent rather than divergent evolution. © 2018 The Authors. IUBMB Life published by Wiley Periodicals, Inc. on behalf of International Union of Biochemistry and Molecular Biology, 70(12):1222–1232, 2018

**Abbreviations**
AAC: ADP/ATP carrier
AGC: aspartate/glutamate carrier
APC: ATP‐Mg/P_I_ carriers
CSN: cytoplasmic salt‐bridge network
F_1_F_O_: F_1_F_O_ ATP synthase
LETM1: leucine zipper‐EF‐hand‐containing transmembrane protein1
MCU: mitochondrial calcium uniporter
PiC: mitochondrial phosphate carrier
MSN: matrix salt‐bridge network
SCaMC: short calcium‐binding mitochondrial carrier

## INTRODUCTION: THE MITOCHONDRIAL ATP‐Mg/P_i_ CARRIER

**Figure 1:**
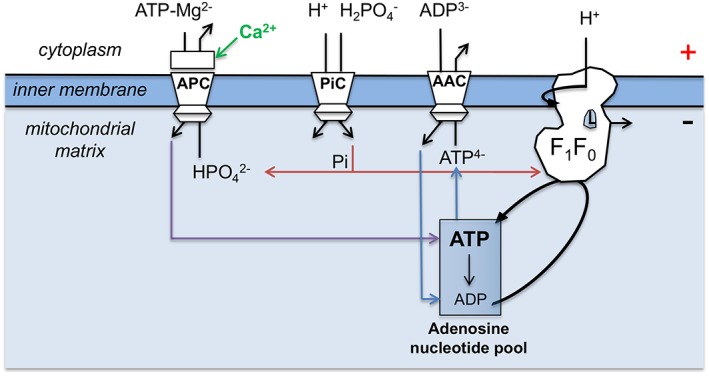
Schematic representation of the function of mitochondrial ATP‐Mg/P_i_ carrier in regulating the adenosine nucleotide pools. Abbreviations: APC; mitochondrial ATP‐Mg/P_i_ carrier, PiC; mitochondrial phosphate carrier, AAC; mitochondrial ADP/ATP carrier, F_1_F_O_; F_1_F_O_ ATP synthase.

In mitochondria, ATP is rapidly synthesised from ADP and phosphate (P_i_) by ATP synthase 1. Newly synthesised ATP in the mitochondrial matrix is exchanged for cytosolic ADP by the mitochondrial ADP/ATP carrier (AAC) 2, 3, 4 in order to maintain high concentrations of ATP in the cytosol to drive energy‐requiring cellular reactions, and to return ADP for the synthesis of ATP (Fig. 1). Since the exchange is equimolar and the substrate specificity is limited to ADP and ATP, the transport activity of AAC cannot change the overall concentrations of adenosine nucleotides in the mitochondrial matrix 2, 3. However, there has to be a mechanism to change these pools in response to division of mitochondria and to changes in energy demands. For this purpose, mitochondria have ATP‐Mg/P_i_ carriers (APC), which transport ATP, with or without magnesium, and ADP in exchange for matrix P_i_ (Fig. 1) 5, 6, 7. This asymmetric exchange can alter the matrix adenosine nucleotide concentrations rapidly, which is fundamental for cellular growth and energy metabolism 8, 9, 10. Furthermore, APC is calcium‐regulated, linking its function to changes in cellular energetic demands through calcium signalling (Fig. 1).

**Figure 2:**
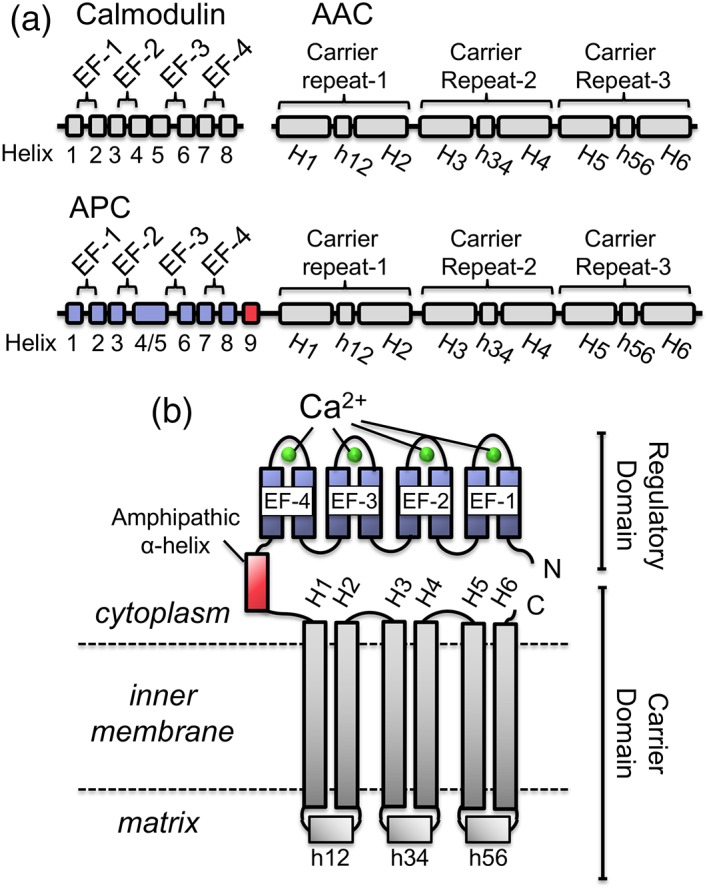
Secondary structure and topology of mitochondrial ATP‐Mg/P_i_ carriers. (A) Secondary structure elements of the mitochondrial ADP/ATP carrier and calmodulin compared to mitochondrial ATP‐Mg/P_i_ carriers. (B) Topology model of mitochondrial ATP‐Mg/P_i_ carriers. Abbreviations: EF, calcium binding EF‐hand domain; H, transmembrane helix.

Among mitochondrial carrier proteins APC is unusual, as it consists of three separate domains. The N‐terminal regulatory domain shares significant sequence identity with four EF‐hand containing proteins, such as calmodulin; human APC1 has ~30% sequence identity with calmodulin, with the seven N‐terminal α‐helices of APC mapping to the eight α‐helices of calmodulin (Fig. 2A) 11, 12. The C‐terminal carrier domain consists of six transmembrane α‐helices and three short matrix α‐helices, and is structurally equivalent to those of mitochondrial carriers in general, but in particular to AAC (~30% sequence identity; Fig. 2A) 13, 14. The linker region between the N‐terminal regulatory domain and the C‐terminal carrier domain does not share any significant sequence homology with either calmodulin or AAC, but consists of a loop containing an amphipathic α‐helix (Fig. 2B) 11, 12.

## APC IDENTIFICATION AND PHYSIOLOGICAL ROLE IN HEALTH AND DISEASE

APC was first described when residual adenosine nucleotide transport in mammalian liver mitochondria was observed after AAC activity was blocked with its specific and potent inhibitor carboxyatractyloside 5, 6, 15. Human APCs were recombinantly expressed and reconstituted into liposomes, and characterised with respect to their transport activity, mode of transport, inhibitor and substrate specificity (ATP, ADP, AMP, ATP‐Mg, dATP, dADP, dAMP P_i_ and pyrophosphate), establishing their molecular identity and properties for the first time 6. Another group also reported the genetic identity of human APCs, noted for their EF‐hands, leading to two different naming conventions: APC 6 and SCaMC (for short calcium‐binding mitochondrial carrier) 16. They have also been assigned to the solute carrier family SLC25 17. In total, there are three human APC isoforms, which have all three domains: APC1 (SCaMC1/*SLC25A24*), APC2 (SCaMC3/*SLC25A25*) and APC3 (SCaMC2/*SLC25A23*), each of which has several possible splicing variants 6, 18, 19 [for a review see ref. 17]. There is also SCaMC3‐like (*SLC24A41*), which appears to function as a calcium‐independent ATP‐Mg/P_i_ carrier, as it lacks the N‐terminal regulatory domain 20.

Broadly, the role of APC is to replenish or deplete the pool of adenosine nucleotides in the mitochondrial matrix in response to high‐ or low‐cellular energetic demands, respectively. The effect of altering the matrix adenosine nucleotide pool in mammalian mitochondria has not been fully resolved. However, oxidative phosphorylation, gluconeogenesis, ureogenesis, mitochondrial protein import, mitochondrial protein folding, mitochondrial DNA transcription, mitochondrial translation and mitochondrial DNA maintenance are all pathways that are influenced by the size of the adenosine nucleotide pool 19, 21.

In *Saccharomyces cerevisiae*, knockout of the APC orthologue (Sal1p) leads to higher rates of spontaneous petite formation 22, presumably because the absence of Sal1p leads to the destabilisation and loss of mitochondrial DNA. A double knockout of both the yeast ADP/ATP carrier AAC2 and Sal1p genes renders yeast non‐viable 7, 23. The impact of APC2 (SCaMC3/*SLC25A23*) knockouts have been studied in mouse liver 9, mouse neuronal tissue 10, 24 and that of an APC3 (SCaMC2/*SLC25A25*) knockout in a mouse model 25. These studies highlight the requirement for adenosine nucleotide movement in and out of mitochondria for respiratory function and reveal a putative further role in the protection of cells under stress‐conditions by reducing calcium‐induced permeabilisation of the mitochondrial inner membrane 26. In a pathophysiological role, overexpression of APC1 can assist cancer cells to evade typical cell death pathways by increasing calcium precipitation in the mitochondrial matrix, reducing calcium‐induced permeabilisation of the mitochondrial inner membrane 27, 28. Conversely, point mutations in human APC1 (SCaMC1/*SLC25A24*) leading to an Arg217His or Arg217Cys substitution have been linked to severe forms of mitochondrial disease related to bone development (Gorlin–Chaudhry–Moss syndrome and/or Fontaine syndrome) 29, 30.

## THE EF‐HAND‐CONTAINING REGULATORY DOMAIN OF APC

**Figure 3:**
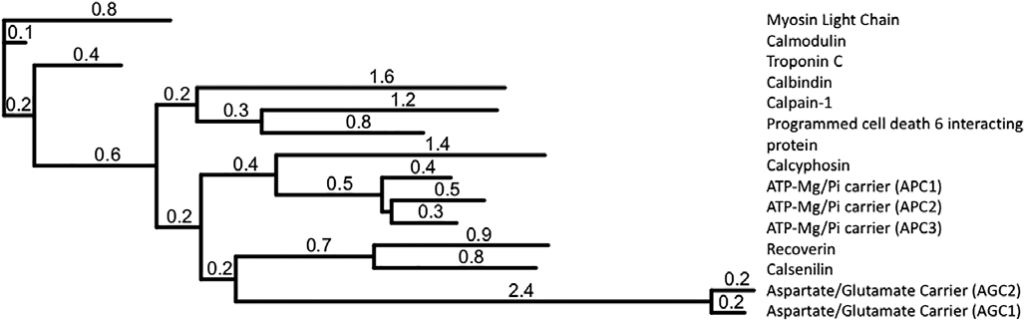
Dendogram of human EF‐hand containing proteins that contain at least two pairs of EF‐hands. Values attached to each branch represent relative sequence deviation. Analysis carried out using Phylogeny.fr 31 and dendogram drawn using iTOL 32.

EF‐hands usually form functional pairs, and the regulatory domain of APC has two pairs. As shown by the phylogenetic analysis of human proteins with at least two EF‐hand pairs (Fig. 3), APC regulatory domains cluster in their own clade. APC regulatory domains share a common branch point with the first four EF‐hands of the mitochondrial aspartate/glutamate carrier (AGC) regulatory domains, which are in a clade of S100‐like EF‐hand proteins together with recoverin and calsenilin. Calcyphosin is the only protein that clusters close to APC regulatory domains, implying an evolutionary link. Furthermore, it is clear that calmodulin is one of the earliest four EF‐hand containing proteins to have arisen. For an extensive review on the evolution of EF‐hand proteins, please refer to ref. 33.

**Figure 4:**
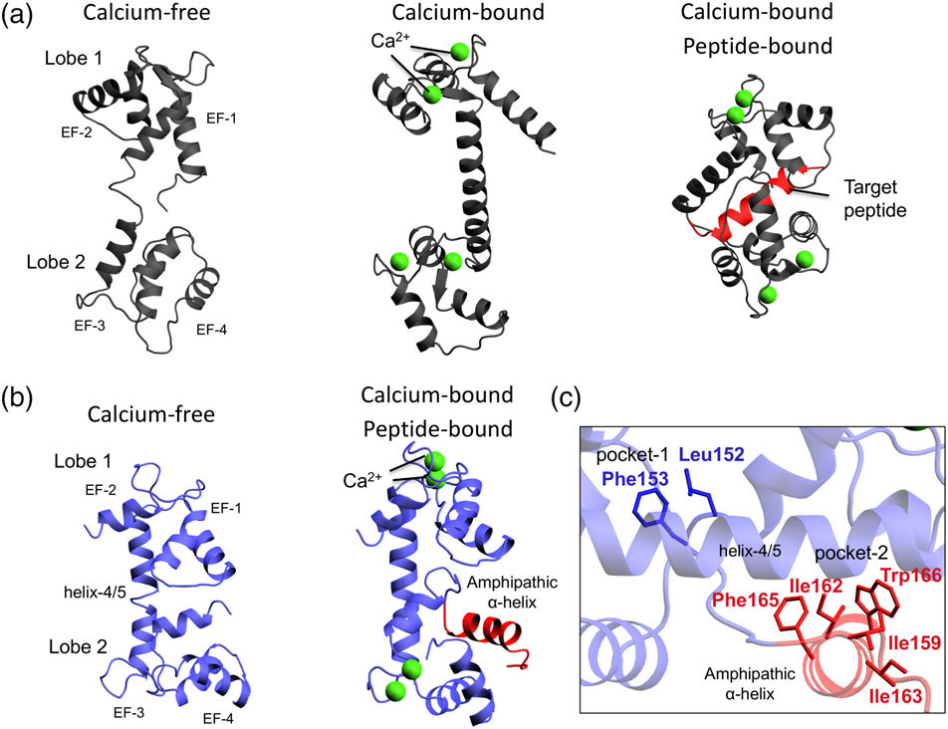
Comparison of calmodulin and regulatory domain structures of mitochondrial ATP‐Mg/P_i_ carriers. (A) Calcium‐free, calcium‐bound and peptide‐bound calmodulin structures (PDB: 1CFD, 1CLL and 1VRK, respectively) 36, 39, 40. (B) Model of the calcium‐free and structure of the calcium‐bound regulatory domain (PDB: 4ZCU) of mitochondrial ATP‐Mg/P_i_ carriers. (C) Detailed view of the residues involved in the interaction between the hydrophobic pockets of the APC regulatory domain and the amphipathic α‐helix of the linker loop domain.

Although the APC regulatory domain appears to be distantly related to calmodulin in evolution, the key structural features of calmodulin are similar to those of APC regulatory domains. Furthermore, the structure and mechanism of calmodulin has been extensively characterised 34, 35, 36, 37, 38 and is the archetypal EF‐hand containing calcium‐binding protein. Therefore, we use calmodulin as a basis to understand the mechanism of APC regulatory domains. Calmodulin is composed of four EF‐hand repeats, where EF‐hands 1 and 2 are paired to form the first lobe and EF‐hands 3 and 4 are paired to form a second lobe. In calmodulin there is a flexible linker between the two lobes. The proposed mechanism for calmodulin‐target interaction suggests this flexible linker allows the lobes to engage target peptides by wrapping around them as observed in crystal structures (Fig. 4A) 36, 39, 40.

There have been several crystal structures of human APC1 regulatory domain in calcium‐bound states (PDBs: 4N5X, 4ZCU, 4ZCV), solved by different approaches, which have confirmed a calmodulin‐like domain architecture 11, 12. In these structures, calcium ions are bound to each of the four EF‐hand motifs (Fig. 4B). In contrast to calmodulin, there is no flexible linker between the exiting α‐helix of EF‐hand 2 and the entering α‐helix of EF‐hand 3. Instead, the helices are fused together, creating one long α‐helix 4/5, meaning that the lobes are rigidly locked in their relative orientations, so unable to wrap around the amphipathic α‐helix of a target protein as calmodulin has been proposed to do (Fig. 4B) 11, 12.

Each EF‐hand of calcium‐bound APC regulatory domain displays an ‘open’ antiparallel arrangement of α‐helices (L‐shape arrangement), equivalent to that of calcium‐bound calmodulin structures (Fig. 4B) 34, 35, 36, 37, 38. Therefore, the hydrophobic core of each lobe is exposed, which in calmodulin allows its target peptides to bind into the hydrophobic binding pockets formed in each lobe 41 (Fig. 3A). In the regulatory domain of human APC, the equivalent hydrophobic pocket of lobe one is ‘capped’ by conserved residues Leu152 and Phe153 at the C‐terminal end of the domain on the final helix of EF‐hand 4, which form hydrophobic interactions with conserved residues of the hydrophobic pocket of EF‐hand pair 1 and 2 (pocket‐1, Fig. 4C). The APC regulatory domain structures also include the linker‐loop domain with the amphipathic α‐helix. The amphipathic α‐helix is bound to the hydrophobic pocket of lobe two, with residues Ile159, Ile162, Ile163, Phe165 and Trp166 forming hydrophobic interactions with conserved residues in the pocket of EF‐hand pair 3 and 4 (pocket‐2, Fig. 4C) 11, 12.

There are no crystal structures of the calcium‐free state of the APC regulatory domain, but, based on bioinformatics analysis and modelling based on other EF‐hand structures, a comparative structure has been obtained (Fig. 4B) 12. In this state, the EF‐hands adopt a closed conformation (U‐shape arrangement) 39, 42, 43 and consequently α‐helices 6 and 7 change their relative orientations, leading to the release of the amphipathic α‐helix from the second hydrophobic pocket (Fig. 4B), similar to the release of signal peptides from the calcium‐free state of calmodulin.

## RESPONSE TO CALCIUM BY DIFFERENT APC ORTHOLOGUES

**Figure 5:**
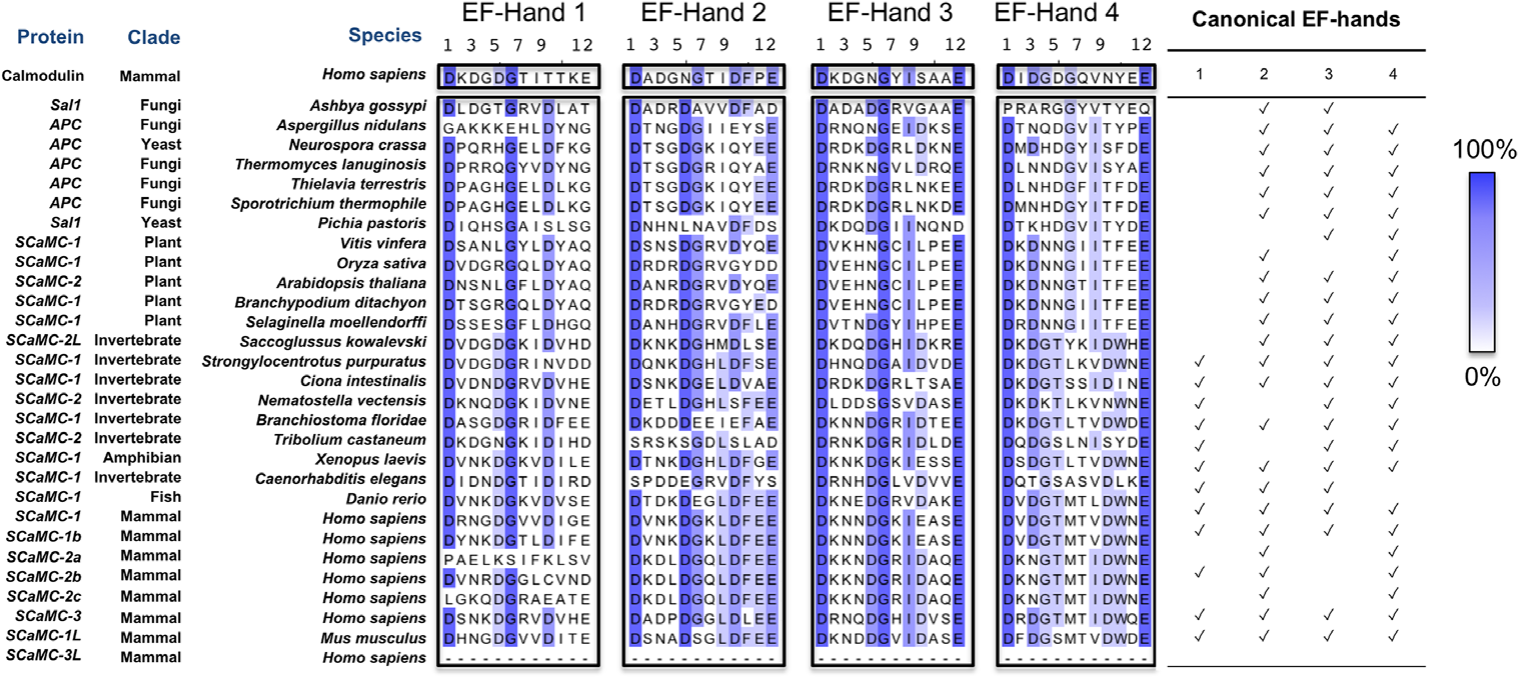
Sequence alignments of EF‐hands in the regulatory domain of mitochondrial ATP‐Mg/P_i_ carriers from mammal, invertebrates, plants and fungi. Residues are coloured according to the percentage of residues in each column that agree with the consensus sequence as in the key. Only the residues that agree with the consensus residue for each column are coloured. Canonical EF‐hands are as predicted by agreement with the PROSITE pattern for calcium binding EF‐hands. SCaMC‐3L does not have an N‐terminal domain. The names of the proteins are based on the NCBI database entries.

Sequence alignments provide evidence that the architecture of the APC regulatory domain is conserved across vertebrates, invertebrates, plant and fungi, in most cases having four EF‐hands (Fig. 5). The consensus motif for a calcium‐binding EF‐hand has been defined by PROSITE as: D‐{W}‐[DNS]‐{ILVFYW}‐[DENSTG]‐[DNQGHRK]‐{GP}‐[LIVMC]‐[DENQSTAGC]‐x(2)‐[DE]‐[LIVMFYW]). Deviations from this consensus, particularly at positions 1 and 12 of the EF‐hand calcium‐binding loop may indicate an inability to bind calcium (Fig. 5). Not all of the EF‐hands are canonical for calcium binding in each of the APC orthologues, as summarised in Fig. 5. For example, APC1 and APC3 from mammals are predicted to bind calcium in all four of their EF‐hands, but the APC2 splice variants have a lysine at position 3 of the motif instead of the consensus aspartate or asparagine residue in EF‐hand 3, indicating decreased or absent affinity for calcium.

A putative reason for these differences is to respond to different levels of calcium stimuli. Indeed, calcium signalling does not have the same physiological role between vertebrates, invertebrates, plant and fungi. Furthermore, humans have an array of different isoforms and splice variants, displaying a complex and overlapping tissue‐specific expression profile. Therefore, calcium concentrations required to stimulate adenosine nucleotide transport are different in each case, because the affinity of EF‐hands for calcium binding has been adapted through specific mutations. The calcium concentration for half‐maximal activity has been estimated to be 15–30 μM calcium for yeast APC 7, 44 180 μM calcium for human APC 45 and 0.2–0.8 μM calcium for *Arabidopsis thaliana* APC 46. Calcium affects the *V*_max_ but not the *K*_m_ for substrate transport in APC, suggesting a non‐competitive mechanism of calcium regulation 44, 45, 46, 47.

Relative to calcium activation of the AGC (from rat liver), which has been measured at around 300 nM 48, 49, APC has a requirement for higher calcium concentrations. The calcium sensitivity of AGC is closer to the calcium activation of other high‐sensitivity calcium sensors in the cell, such as calmodulin, which has a nanomolar affinity for calcium 50. Therefore, APC is a low‐sensitivity calcium sensor, which is not unusual as proteins, such as troponin C 51 and the mitochondrial calcium uniporter (MCU) 52, also have calcium binding constants in the micromolar range.

## THE AAC‐LIKE CARRIER DOMAIN OF APC

**Figure 6:**
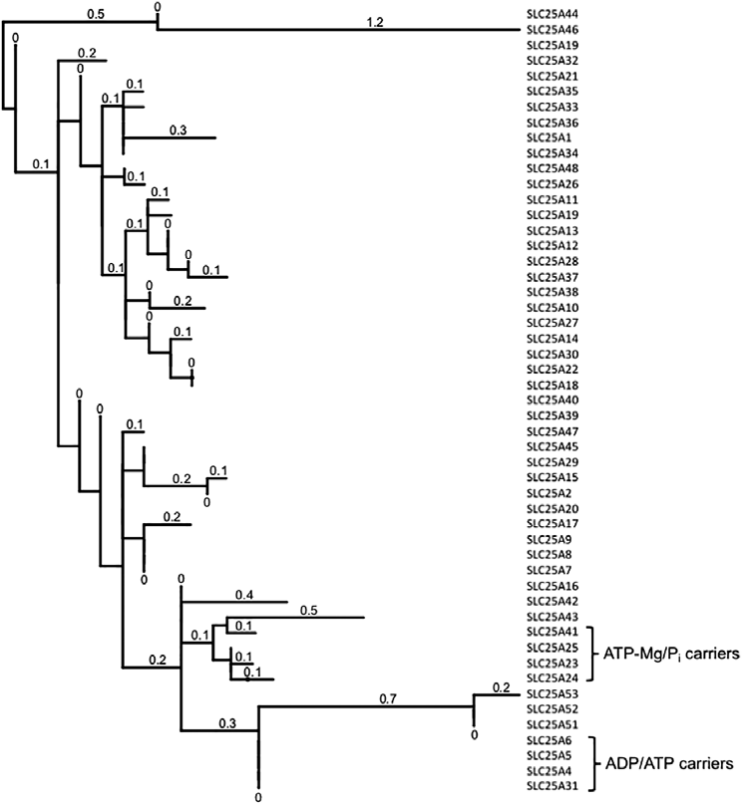
Dendogram of human mitochondrial carriers (solute carrier family 25). Values attached to each branch represent relative sequence deviation. Analysis carried out using Phylogeny.fr 32 and dendogram drawn using iTOL 33.

The carrier domains of APC and AAC share significant sequence identity (33% between human APC1 and human AAC1). Furthermore, phylogenetic analysis of all human mitochondrial carriers shows that APCs and AACs cluster closely, with a common branch point in evolution (Fig. 6). Members of the mitochondrial carrier family have characteristic three homologous sequence repeats 53. The atomic structures of bovine 13 and yeast 14 AAC, inhibited by carboxyatractyloside, demonstrated that each of the three domains fold into two transmembrane α‐helices, connected by a short α‐helix on the matrix side. In the carboxyatractyloside‐inhibited state, the six transmembrane α‐helices are arranged to provide access to the putative central substrate binding site from the cytosolic side.

Mitochondrial carriers have been proposed to function according to a ‘single binding centre gated pore mechanism’ 4, which is in essence an alternating access mechanism 14, 54, 55. Two salt bridge networks have been identified that form part of the gates at the cytoplasmic and matrix side of the carriers that regulate access to the central substrate binding site. The matrix network consists of the charged residues of the threefold pseudo‐symmetrical Px[DE]xx[KR] motif on transmembrane α‐helices H1, H3 and H5 56, forming ionic interactions when the carrier is in the cytoplasmic state 13, 14. The cytoplasmic network is formed by the charged residues of the threefold pseudo‐symmetrical [FY][DE]xx[RK] motif on transmembrane α‐helix H2, H4 and H6 57, which also form ionic interactions in the transport cycle 14, 55, 58.

## SALT‐BRIDGE NETWORKS IN THE APC CARRIER DOMAIN

Some mitochondrial carriers, such as AAC, are strict exchangers of substrates 3. Others display uniport activity, for example, the phosphate and glutamate carriers 59, 60. This feature of the mitochondrial carriers relates to the relative interaction energies of the cytoplasmic and matrix salt‐bridge networks, and the binding energy of transported substrates 57. In the absence of atomic structures of these carriers in different states, it is not possible to quantify the interaction energies of these networks accurately. However, in general, the interaction energies of salt bridges are in the range of 20–40 kJ/mol, whereas hydrogen bonds and cation–π interaction energies are in the range of 8–29 and 8–16 kJ/mol, respectively. In contrast, van der Waals interactions have relatively weak interaction energies of ~1 kJ/mol. To get a rough estimate of the interaction energy of the network a semi‐quantitative measure was introduced, which assigns a value of 1.0 to each potential salt bridge, a value of 0.5 to each potential hydrogen bond or cation–π interaction, and a value of 0.0 to a potential van der Waals interaction 57. The values are then added together to get a rough estimate of the overall interaction energy of the network. This analysis has led to the hypothesis that strict exchangers have equally strong networks for both the matrix and cytoplasmic salt‐bridge network, whereas net importers have a strong matrix network and a weak cytoplasmic network, which is supported by computational analysis 57, 58.

**Figure 7:**
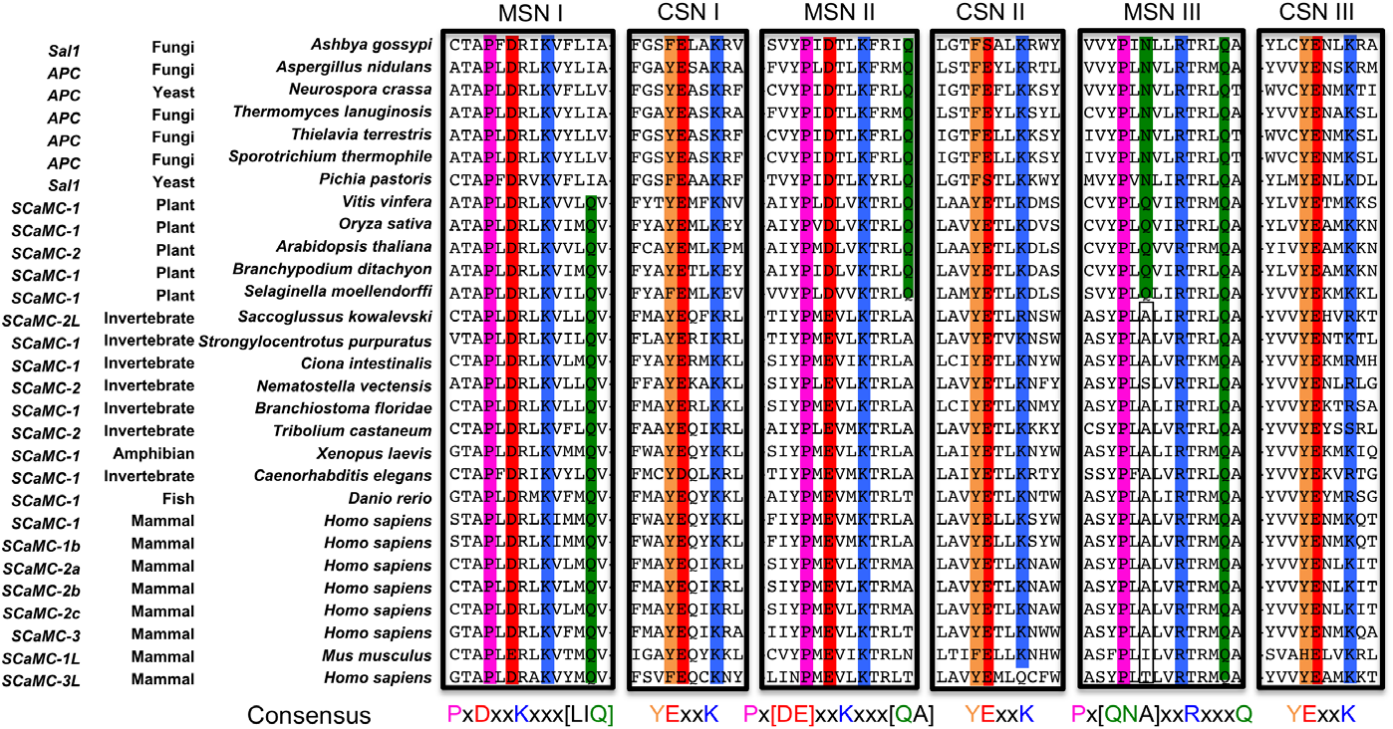
Sequence alignments of salt‐bridge networks in mitochondrial ATP‐Mg/P_i_ carriers. Sequence motifs are highlighted and coloured according to residue properties (helix‐breaking, magenta; negatively charged, red; positively charged, blue; hydrophilic, green; aromatic, orange) 61. MSN; matrix salt‐bridge network, CSN; cytoplasmic salt‐bridge network. The names of the proteins are based on the NCBI database entries.

**Figure 8:**
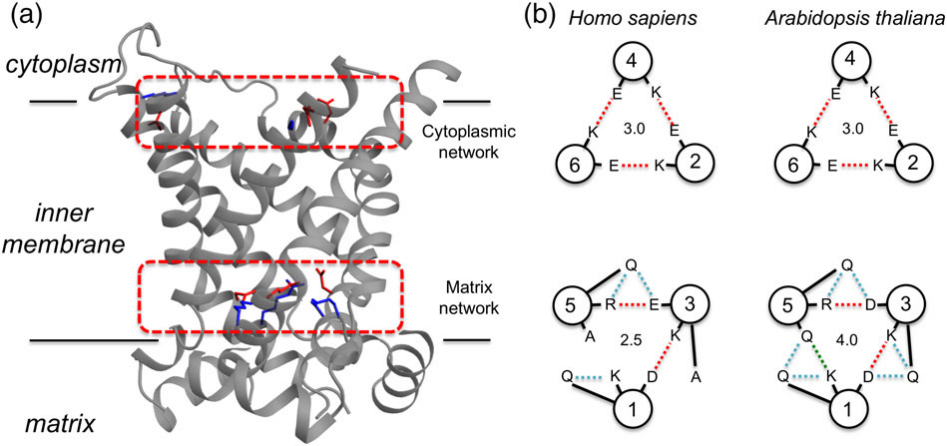
Salt‐bridge networks in mitochondrial carrier domains. (A) Positions of the unformed cytoplasmic salt‐bridge network and the formed matrix salt‐bridge network in bovine AAC (PDB: 1OKC). (B) Schematic representations of the cytoplasmic and matrix salt‐bridge networks in human (isoform 1) and *A. thaliana* APC orthologues. The semi‐quantitative interaction energies of the networks are indicated. Salt‐bridge interactions are represented by red dashed lines, whereas hydrogen bonds are displayed by green or cyan dashed lines for inter‐helical bonds or ‘glutamine braces’, respectively.

The cytoplasmic salt‐bridge network of APC is highly conserved across all domains of life (Fig. 7), with all three repeats of the motif conforming to the consensus sequence of Y[ED]xx[RK], providing the potential to form three salt‐bridge interactions, giving a semi‐quantitative value of 3.0 (Fig. 8B). The matrix salt‐bridge network of APC in plant and fungal orthologues share the consensus sequence of Px[ED]xx[RK] for the first two repeats of the motif. However, in the final motif on the fifth transmembrane helix, the negatively charged residue on H5 is replaced with either an asparagine residue in fungal APCs or a glutamine residue in plant APCs. Therefore, the matrix salt‐bridge network has the potential to form two salt‐bridge interactions and one hydrogen bond interaction, giving a total of 2.5. In plant orthologues there are three glutamine residues and in fungal orthologues two glutamine residues that form braces with the residues of the matrix salt‐bridge network, which adds to the total interaction energy of the network 14, providing a total of 4.0 and 3.5, respectively (Fig. 8B). The matrix salt‐bridge network of APC in members of the *animalia* kingdom also share the consensus sequence of Px[ED]xx[RK] for the first two repeats of the motif, however, in the final repeat, the negatively charged residue on H5 is replaced with an alanine residue. This network has the potential to form only two salt‐bridge interactions, but it has two out of the three possible glutamine braces, providing at total of 2.5 (Fig. 8B). There could be other interactions in the cytoplasmic network that have not yet been appreciated. However, in the current assessment the strength of the cytoplasmic and matrix salt‐bridge networks in APC are roughly equal, implying that APCs are strict exchangers of substrates, agreeing with experimental evidence that APC does not catalyse a significant uniport activity 6, 45.

## SUBSTRATE BINDING AND TRANSPORT IN THE APC CARRIER DOMAIN

Both APC and AAC are transporters of adenosine di‐ and tri‐nucleotides 62, but APC catalyses an electroneutral exchange, whereas AAC catalyses an electrogenic exchange 3, 6, 18, 62, 63. Electroneutral exchange implies that ATP is transported together with magnesium or protons in exchange for phosphate, as both exchanged species would then carry the same charge 6, 18, 19. The affinity of APC for ATP has been observed in the range of 30–300 μM for a number of different orthologues 6, 44, 45, 46, 64. This affinity is 10 to 100‐fold less than the affinity of AAC for ATP, which is in the range of 2–20 μM 44, 65, 66, 67, 68.

**Figure 9:**
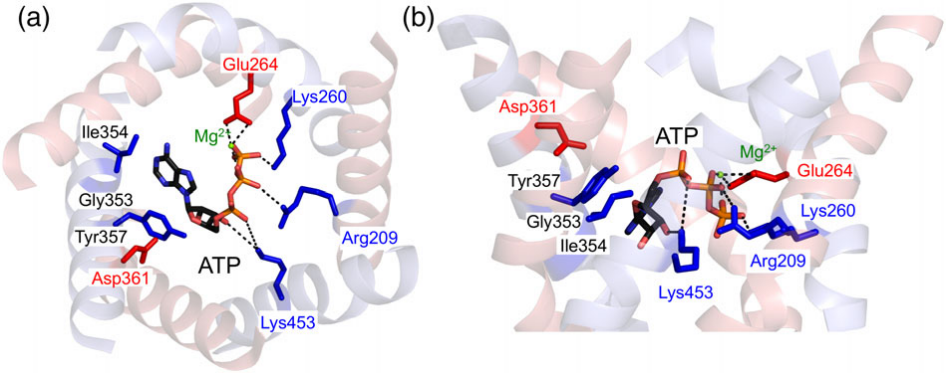
Proposed binding arrangement of ATP‐Mg in the carrier domain of the mitochondrial ATP‐Mg/P_i_ carrier. View from the (A) cytoplasm and (B) from the membrane of a comparative model of human APC1 based on bovine AAC1 (PDB: 1OKC) 13 to highlight the conserved residues within the proposed binding site. ATP‐Mg, represented as sticks, was manually docked in analogy to ADP binding to AAC1 62, 70. Odd‐numbered and even‐numbered helices are coloured blue and red, respectively. Residues that are common to both AAC and APC have been coloured blue, and those uniquely found in APC are coloured red. Residues are numbered according to their amino acid positions in the human APC1 sequence.

The residues proposed to be involved in substrate binding in APC and AAC are very similar. In human APC1 they consist of Gly353, lle354 and Tyr357, which might be involved in adenosine binding, and Arg209, Lys260 and Lys453, which might be involved in binding of the phosphate groups (Fig. 9) 57, 69. In addition, two highly conserved, negatively charged residues, Glu264 and Asp361, are close to the proposed nucleotide‐binding site and are absent from AAC (Fig. 9) 57. These residues could be important for the binding of magnesium during ATP‐Mg transport 57, 69.

## THE MECHANISM OF CALCIUM REGULATION IN APC

**Figure 10:**
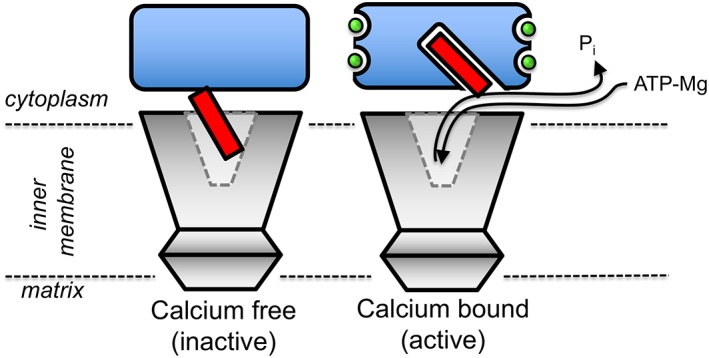
Schematic representation of the locking pin mechanism of calcium regulation of the mitochondrial ATP‐Mg/P_i_ carrier. Analysis of mutants with thermostability assays has shown that in the absence of calcium the carrier domain is stabilised by binding of the amphipathic α‐helix 45, a state that is inhibited for substrate transport. Structural analysis has shown that in the calcium‐bound state the amphipathic α‐helix is bound to the regulatory domain 11, 12, freeing the carrier domain of the amphipathic α‐helix and allowing substrate transport.

To understand how the AAC‐like carrier domain and the EF‐hand‐containing regulatory domain function together to provide calcium‐regulated adenosine nucleotide transport, an understanding of the overall position of the three APC domains relative to one another is required. In the absence of an experimental structure, a model of the protein has been pieced together using evolution coupling and structural data 45. This model, coupled with all available biophysical characterisation data, suggests that the carrier and regulatory domains move independently of each other and interact only via the linker‐loop region 45. In the proposed *locking pin mechanism*, the amphipathic α‐helix is released from the regulatory domain when the calcium levels are low, leading to closure of the hydrophobic pockets and to its binding with residues of the cavity in the carrier domain, inhibiting the transporter domain. In the calcium‐bound state, the hydrophobic pockets open, providing a higher‐affinity binding site for the amphipathic α‐helix, removing it from the carrier domain and allowing the transport of substrates (Fig. 10).

In support of the idea that the amphipathic α‐helix is the key element, a number of point mutations were tested for their effect on regulatory function 45, identifying a triplet of highly conserved charged residues named the ERD clasp (Glu88, Arg148 and Asp149 in human APC1), which form a salt‐bridge interaction that stabilises the binding of the amphipathic α‐helix in the calcium‐bound regulatory domain. Mutation of any one of these residues shifts the protein toward a constitutively inactive, inhibited state, even in the presence of calcium. Conversely, mutations of several residues of the amphipathic α‐helix or the carrier domain (such as Asp176, Lys167, Glu183 and Arg291 in human APC1) had the opposite effect and shifted the behaviour of the carrier towards a state that was active even in the absence of calcium, most likely because they are required for the binding of the amphipathic α‐helix to the carrier domain 45.

## COMPARISON OF THE APC MECHANISM WITH OTHER CALCIUM‐REGULATED MEMBRANE PROTEIN MECHANISMS

Normally, EF‐hand mechanisms involve the binding of an amphipathic α‐helix in a target polypeptide to a calcium‐bound EF‐hand, propagating downstream signalling events 70. This is a common mechanism in the regulation of ion channels such as SK potassium channels 71. In the SK potassium channel, lobe 2 of calmodulin binds to the channel constitutively, whereas lobe 1 does not bind in the absence of calcium. Once calcium‐bound, calmodulin changes its conformation and interacts with its target in the protein (the S4‐S5 linker), resulting in channel pore opening 71.

The regulatory mechanism in APC shares features of this mechanism, but differs also, as the regulatory amphipathic α‐helix and EF‐hands are part of the same polypeptide chain. There are other examples of calcium‐regulated membrane proteins where the EF‐hands are part of the same polypeptide chain, such as the mitochondrial leucine zipper‐EF‐hand‐containing transmembrane protein1 (LETM1) 72, subunits of the mitochondrial calcium uniporter (MICU1) 73 and ryanodine receptors 74. In the case of LETM1 and MICU1, the EF‐hand mechanisms in these proteins have not been fully resolved. In the ryanodine receptor, there is a single EF‐hand per protomer of the tetrameric protein, an unusual arrangement as EF‐hands normally function as pairs. It has been proposed that the conformational change from a U‐shape to and L‐shape in the ryanodine receptor EF‐hand upon calcium binding propagates a larger structural transition, resulting in an active channel 74.

The protein that shares the most similarities with APC in terms of its combination of domains and its mechanism of action is AGC. Like APC, AGC displays a calcium‐dependent stimulation of activity in its carrier domain 75, 76, 77, 78 when calcium binds to its EF‐hand containing N‐terminal regulatory domain 79. However, there are distinct differences in the proposed regulatory mechanism of AGC from APC. The mechanism in AGC involves rotation of the mobile unit of EF‐hands 1–2 against the static unit of EF‐hands 4–8 of the regulatory domain. This conformational change regulates substrate transport by closing or opening access to the carrier domain 79.

## CONVERGENT EVOLUTION OF CALCIUM REGULATION IN APC AND AGC

The combination of different domains in APC represents an interesting example of evolution. Domains from different evolutionary backgrounds have been combined in order to regulate substrate transport. There are other examples of EF‐hand‐containing proteins that have this type of ‘self‐sequestered’ interaction 80, 81, 82, 83, but only the AGC has a similar combination of domains 79. Even so, APC and AGC do not share significant sequence similarity in their regulatory domains; APC has canonical EF‐hand motifs like calmodulin, whereas AGC contains S100‐like calcium‐binding motifs 12, 79. In addition, the AGC regulatory domain contains a dimerisation interface for the formation of homo‐dimers 79, whereas APC is monomeric 12. Furthermore, in AGC the carrier domain is inserted between the EF‐hands and the target α‐helix, whereas in APC the target α‐helix is inserted between the EF‐hands and the carrier domain. Therefore, it appears that APC and AGC have both evolved the ability to regulate substrate transport in response to changes in calcium concentration using very different mechanisms, an intriguing example of convergent evolution.

## CONCLUSION

The fusion of two proteins by recombination is a faster mechanism for producing protein with new functionality than *de novo* evolution. APC represents an example of this type of evolution, combining an archetypal mitochondrial AAC‐like carrier domain and an EF‐hand containing domain to produce calcium‐regulated adenosine nucleotide transport activity. By and large, the regulatory domain and the carrier domain function the same way as their closest relatives, and it is the addition of linker domain with the amphipathic α‐helix that has allowed self‐regulation. This type of ‘self‐sequestration’, where the target peptide is part of the same polypeptide chain, is not uncommon in EF‐hand containing proteins. For example, the protein calcyphosin shares domain organisation with the regulatory domain of APC 83, and it is likely that a ‘self‐sequestering’ EF‐hand protein like this was the precursor for the APC regulatory domain. This means that in its evolution, APC required relatively few sequence changes in order to adapt it for calcium‐regulated adenosine nucleotide transport activity. Aside from its interesting ancestry, APC activity plays an important role in the function of cells in health, whereas it causes severe human pathophysiology in dysfunction. A deeper understanding of its mechanism is worthy for future research.

## CONFLICT OF INTEREST

The authors declare no conflict of interests.
